# Increased Brain White Matter Axial Diffusivity Associated with Fatigue, Pain and Hyperalgesia in Gulf War Illness

**DOI:** 10.1371/journal.pone.0058493

**Published:** 2013-03-20

**Authors:** Rakib U. Rayhan, Benson W. Stevens, Christian R. Timbol, Oluwatoyin Adewuyi, Brian Walitt, John W. VanMeter, James N. Baraniuk

**Affiliations:** 1 Georgetown University Medical Center, Department of Medicine, Division of Rheumatology, Immunology and Allergy, Washington, DC, United States of America; 2 Georgetown University Medical Center, Department of Neurology, Center for Functional and Molecular Imaging, Washington, DC, United States of America; 3 Washington Hospital Center; Division of Rheumatology, Washington, DC, United States of America; Hangzhou Normal University, China

## Abstract

**Background:**

Gulf War exposures in 1990 and 1991 have caused 25% to 30% of deployed personnel to develop a syndrome of chronic fatigue, pain, hyperalgesia, cognitive and affective dysfunction.

**Methods:**

Gulf War veterans (*n = *31) and sedentary veteran and civilian controls (*n = *20) completed fMRI scans for diffusion tensor imaging. A combination of dolorimetry, subjective reports of pain and fatigue were correlated to white matter diffusivity properties to identify tracts associated with symptom constructs.

**Results:**

Gulf War Illness subjects had significantly correlated fatigue, pain, hyperalgesia, and increased axial diffusivity in the right inferior fronto-occipital fasciculus. ROC generated thresholds and subsequent binary regression analysis predicted CMI classification based upon axial diffusivity in the right inferior fronto-occipital fasciculus. These correlates were absent for controls in dichotomous regression analysis.

**Conclusion:**

The right inferior fronto-occipital fasciculus may be a potential biomarker for Gulf War Illness. This tract links cortical regions involved in fatigue, pain, emotional and reward processing, and the right ventral attention network in cognition. The axonal neuropathological mechanism(s) explaining increased axial diffusivity may account for the most prominent symptoms of Gulf War Illness.

## Introduction

Over 25% of the 697,000 veterans deployed to the 1990–1991 Persian Gulf War and 15% of nondeployed veterans have developed a symptom complex of widespread pain, fatigue, headache, gastrointestinal, bladder, and other “functional” nociceptive and interoceptive complaints [1]–[4]. Gulf War veterans were exposed to a wide variety of exposures that include binary nerve agents, acetylcholinesterase inhibitors, organophosphates, other pesticides and herbicides that may have initiated their symptom complex [3]. This syndrome has been termed Gulf War Illness (GWI). An initial analysis defined these subjects as Chronic Multisymptom Illness (CMI) [1], [2] based on ≥2 complaints of (i) fatigue, (ii) musculoskeletal or (iii) mood and cognitive dysfunction for ≥6 months [2]. Deployed Gulf War veterans met criteria for Chronic Fatigue Syndrome (CFS) (odds ratio = 40.6) and Fibromyalgia (FM) (odds ratio = 2.32) indicating extensive symptom overlap [5], [6], [7]. All of the veterans who met CMI criteria in this study also met CFS criteria, and 52% met FM criteria.

A striking clinical observation in our GWI subjects has been that their chronic pain and fatigue fluctuate in parallel [1]. Fatigue represents an increase in the presumed effort required to perform usual activities, and is not the same as tiredness or sleep deprivation [5]. Pain is the subjective sensory and affective perception reported by subjects in response to a potentially harmful stimulus [8]. Pain and fatigue have been associated with structural and functional alterations of cortical regions in chronic regional pain syndrome, penetrating brain injury, migraine, irritable bowel syndrome and chronic pelvic pain [9]–[13].

Efforts have been made to define both fatigue and pain in terms of functional neurobiology [14], [15]. Activity in the orbitofrontal cortex (OFC) has been associated with both the fatigue sensation induced by a prolonged cognitive task and with accurate discrimination between painful and non-painful perceptions [16], [17]. The OFC has strong reciprocal projections with the adjacent ventromedial prefrontal cortex (vmPFC). The severity of damage to the vmPFC correlates with fatigue [10]. Further, increased functional connectivity between the right vmPFC, nucleus accumbens and anterior insula, mediated via the inferior fronto-occipital fasciculus (IFOF), contributes to the development and maintenance of chronic pain. [18], [19].

The prefrontal cortex is the only cortical area to receive direct projections from the spinal cord [20]. Central sensitization mechanisms that alter the spinal cord dorsal horn gating of pain transmission lead to increased perceptions of pain after an aversive, physical stimulus such as cutaneous pressure (hyperalgesia) or an innocuous stimulus such as light touch (allodynia) [21], [22]. These molecular events may sensitize prefrontal regions of the brain’s “pain matrix” leading to structural and functional reorganization associated with chronic symptom complaints [23].

Brain dysfunction in CMI may involve changes in white matter integrity. White matter function can be analyzed using diffusion tensor imaging (DTI) which assesses the random Brownian motion and the orientation of water molecules in a strong magnetic field [24]–[27]. Fractional anisotropy (FA) is the most commonly reported index. A decrease in FA indicates loss of white matter integrity [28]. FA is defined as the inverse of the 3 eigenvectors that describe the potential diffusion of water in nerve bundles [28]. The primary eigenvector describes water diffusivity in the direction of the fiber tract and is called the axial diffusivity (AD). Histological information indicates AD assesses axonal function [29]. Diffusion of water perpendicular to axons is defined by two eigenvectors and reported as the radial diffusivity (RD). RD has been associated with demyelination, neuroinflammation with edema or macrophage infiltration [30]–[32]. Mean diffusivity (MD) is the average of the AD and 2 RD eigenvectors. AD, RD, MD and FA can be correlated with subjective and objective outcomes to determine their relationships with tract integrity.

White matter integrity and CMI symptoms have not been investigated. Given the concomitant chronic fatigue and pain, we hypothesized CMI may have significant white matter dysfunction compared to control subjects in tracts connecting the prefrontal areas involved in pain and fatigue. Correlations of fatigue, pain and hyperalgesia with AD and RD of specific white matter tracts were expected to implicate alterations of axonal or dysmyelination processes, respectively, to brain regions responsible for these clinical features.

Two possible outcomes were envisioned. Significant correlations between fatigue, pain, hyperalgesia, and DTI variables for specific tracts that are distinct from control subjects would support the hypothesis that CMI is a disease with characteristic central nervous system pathology with bimodal distribution. Alternatively, these correlations may occur across both the CMI and control populations. This would suggest that CMI represents a highly skewed population distribution selected by symptom severity; any neurologic alterations seen in CMI would be interpreted as the far end of the distribution of a normal physiological process. There is no data regarding white matter integrity in CMI. Our findings advance current knowledge by investigating/integrating objective DTI outcomes into CMI neuropathology.

## Materials and Methods

### Subjects, Ethics Statement and Recruitment

Protocols were approved by the Georgetown University Institutional Review Board and USAMRMC Human Research Protection Office (HRPO #A-15547.0) (clinicaltrials.gov identification number NCT01291758). All participants signed an informed consent. The subject pool was composed of 31 veterans who met CMI and CFS criteria, and 12 sedentary control veterans and civilians not meeting CMI or CFS criteria (Georgetown University IRB #2009–229) All of these subjects completed psychometric questionnaires and physical examinations (*n = *43). For the initial DTI analysis (*n = *51), 8 additional age - matched, healthy sedentary female control civilians(#2010–050 and #2010–356) were recruited. Complete details involving recruitment and retention of all participants can be found in Table B in File S1.

On – line questionnaires (Table B in File S1) assessed an extensive set of psychometric qualities to investigate the distinctions between CMI, CFS and control subjects [33]. The current study focused only on fatigue, pain and hyperalgesia. Other data will be reported elsewhere.

Fatigue was assessed with the ordinal fatigue rating, Chalder fatigue scale and the Multidimensional Fatigue Inventory (MFI) [34], [35]. The ordinal fatigue assessment was anchored with 0 = no complaint, 1 = trivial, 2 = mild, 3 = moderate or 4 = severe intensity [36]–[38]. Inclusion of “trivial” allowed participants to verify complaints that were present but not bothersome enough to warrant treatment and/or other lifestyle changes [39]. Subjective pain perceptions were quantified using the McGill short form with its sensory, affective and total scores [40] and nominal analysis of widespread pain in 4 quadrants and the axial skeleton [7]. Relative disability and quality of life were compared using the Medical Outcomes Survey Short Form 36 (SF-36) [41]. Subjects on medications (analgesics, sedative, tricyclic and other antidepressant drugs) had their medications tapered over a 2 week period.

### Protocol

Upon arrival to the Georgetown University Clinical Research Unit, participants reviewed and signed their informed consent forms. All subjects had history and physical examinations to assess CMI [2], CFS [5], and fibromyalgia criteria [7], [42]. The protocol also included clinical assessments, blood tests, dolorimetry [43], and had a tour of the facilities to familiarize themselves with the fMRI and other equipment.

Hyperalgesia in fibromyalgia has traditionally been ascertained by tenderness to manual thumb pressure of about 4 kg at ≥11 of 18 tender points [7]. We adapted this concept by pressing a pressure strain gauge dolorimeter at the 18 sites at a rate of 1 kg/sec [43]. Subjects were instructed that they were in control of the pressure, and to report the point when the pressure sensation switched to become painful. The average of the dolorimetry pressures has been used as a measure of systemic hyperalgesia [43].

### Diffusion Tensor Imaging (DTI)

Data was acquired on a Siemens 3T Tim Trio scanner equipped with transmit-receive body coil and commercial 12-element head coil array. Two DTI scans were acquired for each subject with parameters of TE = 101 ms, TR = 7900 ms, FOV = 240 mm, 55 slices, slice resolution = 2.5 mm, voxel size = 2.5×2.5×2.5 mm. For each scan, 5 non-diffusion weighted volumes (b = 0 s/mm^2^) and 30 diffusion-weighted volumes (b = 1000 s/mm^2^) were acquired. For each subject, the two DTI scans were concatenated to increase the signal-to-noise ratio. All MR images were screened for abnormal radiological/structural appearances by a trained technician.

Preprocessing of the individual subject’s DTI data was performed with the TORTOISE (version 1.1.2) processing pipeline [44]. Default settings were used except where noted otherwise. Eddy current distortion and motion correction were applied [45]. Susceptibility-induced EPI distortion correction was performed using the first B0 image as a target for registration [46]. Rigid reorientation was applied to the subject’s diffusion weighted images, bringing them into a common final space as defined by the registered first B0 image. All corrections were performed in the native space of the diffusion weighted images, all transformations were applied in a single interpolation step, and the b-matrix was reoriented appropriately [47]. In preparation to calculate the FA image, the signal standard deviation was calculated with the automatic method option, and then the diffusion weighted images were masked with the masking tool. FA and eigenvalue images were calculated using the iRESTORE algorithm provided with TORTOISE, which is a non-linear least squares method of tensor estimation [48]. Subject specific maps for FA and MD were direct outputs from the TORTOISE program.

The AD maps were the first eigenvalue images, while RD maps were calculated by taking the average of the second and third eigenvalue images. Tract-Based Spatial Statistics (TBSS), which is part of the FSL software package [49], [50] was used to transform the images into common Montreal Neurological Institute (MNI) space. All default settings were used. The subjects’ FA data were imported into TBSS, and then aligned into MNI space using the nonlinear registration tool FNIRT [50], [51], which uses a b-spline representation of the registration warp field [51]. This transformation was subsequently applied to the AD, RD, and MD images. The Johns Hopkins University white-matter tractography atlas was used to create masks to extract mean values for each tract [52]–[54]. Brain extraction was performed, using the brain extraction tool implemented in FSL [55].

### Statistical Analysis

SPSS for Windows version 20 (Armonk, New York) and Microsoft Excel 2007 (Redmond, Washington) were used for database and statistical analysis. Ordinal fatigue, McGill total score and dolorimetry were compared between the 31 CMI and 12 control subjects and reported as means with 95% confidence intervals. Significant differences between groups (*P*≤0.05) were identified by two-tailed unpaired Student’s t-tests or Fisher’s exact tests, with all *P* values corrected for multiple comparisons by Bonferroni corrections and false discovery rate [56]–[58]. Ordinal fatigue, McGill total score and dolorimetry were evaluated by receiver – operator curve (ROC) analysis as predictive measures. Clinical data from the eight control participants recruited under protocols #2010-050 and #2010-356 were not included in this analysis but will be reported separately.

FA, MD, AD, and RD for each tract were correlated in univariate fashion with ordinal fatigue by one - tailed Spearman’s function, and for McGill total pain and average dolorimetry pressures using one - tailed Pearson’s function based upon previous studies [9], [13], [18]. The clinical variables with significant DTI correlates were then assessed by separate step-wise multivariate linear regression analyses that included age and gender to identify significantly associated tracts and clinical features across all subjects. Average white matter diffusivity parameters (FA, MD, AD and RD) were compared between all CMI and control participants (*n = *51) and in 20 white matter tracts by two-tailed unpaired Student’s t-tests. ROC analysis of each tract identified the sensitivity, specificity, area under the curve (AUC), and asymptotic significance of each diffusivity parameter. The thresholds were used to define dichotomous variables for stepwise forward binary logistic regression to predict CMI status.

## Results

### Demographics

All of the veterans recruited met CMI and CFS criteria [2], [5]. 52% of the veterans met criteria for FM [7] (Table A in File S1). The CMI (45.9 yr [43.2 to 48.4] (mean [±95% confidence interval]); 81% male; *n = *31) and control (45.6 yr [41.2 to 50.5]; 55% male; *n = *20) groups had equivalent ages and genders (Table 1).

**Table 1:** Demographics.

	Controls	CMI
**Age**	45.6 yr [41.2 to 50.5]	45.9 yr [43.2 to 48.4]
**Gender**		
Male	11	25
Female	9	6

### Fatigue, Pain and Hyperalgesia

**Figure 1:**
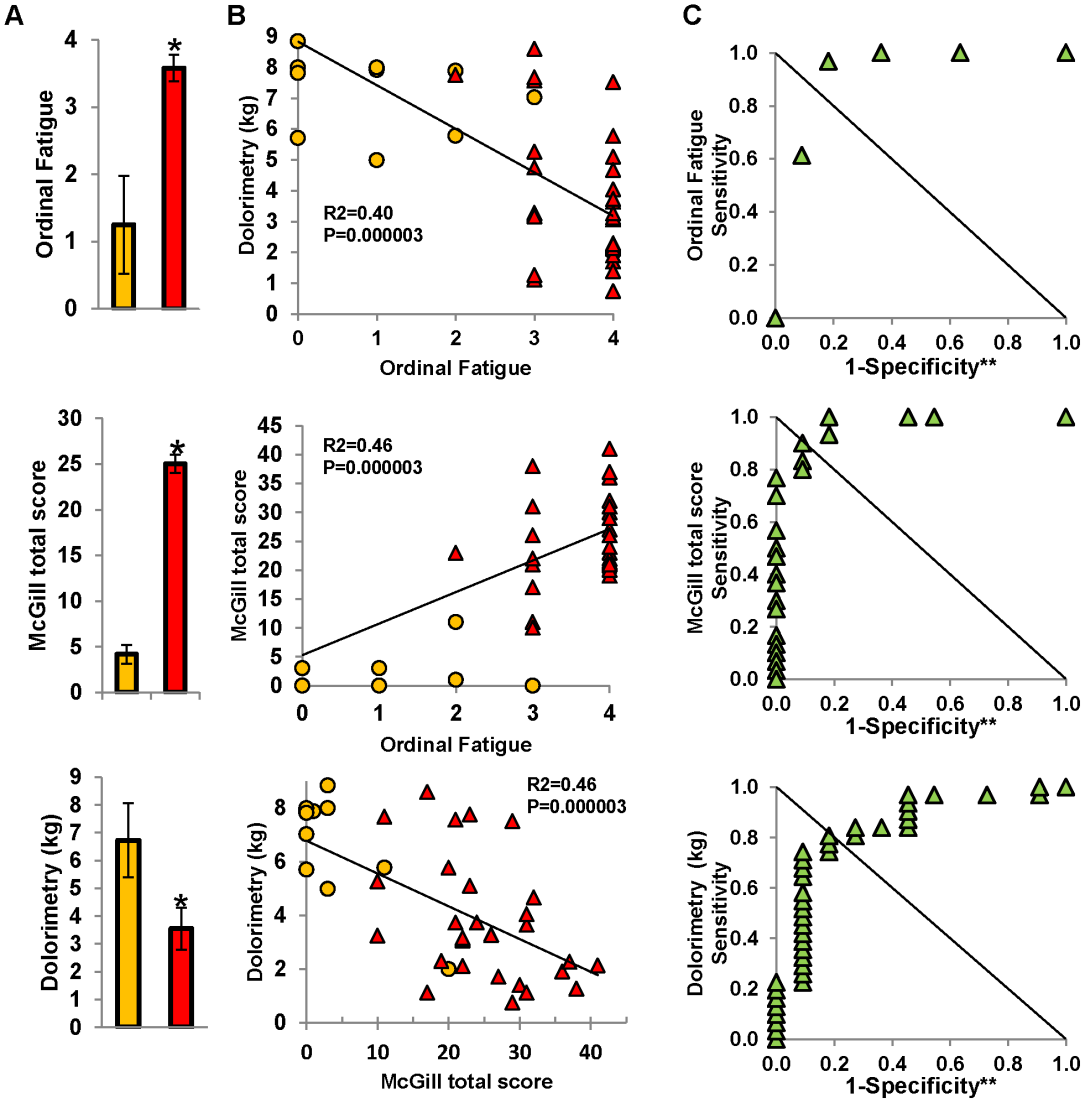
Correlation between McGill total score, mean pain threshold and fatigue. (A) The bar graphs show the magnitudes of the significant differences in ordinal fatigue (* *P = *1×10^−9^), McGill total score (* *P = *1×10^−9^), and pain threshold by dolorimetry (* *P = *6×10^−4^) for control (orange) and CMI (red) groups. (B) Scattergrams from all subjects show the relationships between clinical measures. Dolorimetry pressures negatively correlated with McGill total score and fatigue ratings for control (orange) and GWI (red) subjects. McGill total pain and ordinal fatigue were positively correlated. (C) ROC analysis set the threshold for separation of controls from CMI subjects at 2.5 for ordinal fatigue (specificity = 0.818; sensitivity = 0.968; AUC = 0.915; **P = 0.00005). The threshold for dolorimetry was 5.8 kg (specificity = 0.73; sensitivity = 0.84; AUC = 0.845; **P = 0.0007). McGill total score of 14 separated CMI from controls (specificity = 0.90; sensitivity = 0.91; AUC = 0.973; **P = 4.4×10^−6^). (*P<0.001, FDR corrected; **P<0.05; Asymptotic significance; error bars depict ±95% confidence intervals).

Ordinal fatigue ratings were significantly higher in CMI than control subjects (*P*<0.0001; Figure 1A). This was verified by Chalder’s fatigue scale and less robustly by MFI general fatigue scores (Table 2). CMI subjects had significantly higher McGill affective, sensory and total pain scores than controls (*P*<0.000001; Figure 1A and Table 2). Quality of life assessed by SF-36 was significantly lower in all domains for CMI than controls (*P*<0.000001; Table 3) indicating greater disability in the CMI group.

**Table 2:** Pain, fatigue and tenderness scores.

	Controls (*n* = 12)	CMI (*n* = 31)	*P* values
**Fatigue Instruments**			
Ordinal fatigue rating	1.25 [0.52 to1.98]	3.58 [3.38 to 3.78]	1×10^−9^
Chalder’s total score	11.0 [8.58–13.42]	25.7 [24.3–27.1]	5×10^−13^
MFI general score	11.1 [9.12–13.1]	13.3 [12.7–14.5]	0.058
**McGill Questionnaire**			
Sensory Score	3.75 [0.68 to6.82]	18.41[16.25 to20.58]	8×10^−9^
Affective Score	0.42 [−0.09 to 0.93]	6.62 [5.52 to 7.72]	9×10^−8^
Total Score	4.17 [0.66 to7.67]	25.03 [22.11 to27.96]	1×10^−9^
**Dolorimetry**			
Pain Threshold	6.73 [5.39 to8.07]	3.55 [2.79 to 4.31]	6×10^−4^

Severity of fatigue using a 5 point ordinal scale verified by Chalder’s and MFI general fatigue scores. CMI subjects have significantly decreased systemic pain threshold and higher McGill total and subscale scores. (2-tailed unpaired student’s t-test corrected using FDR, *P*<0.05; Mean [95% Confidence intervals]).

**Table 3:** MOS-SF-36 Quality of Life Domains for CMI and controls.

Domain	Controls (*n* = 12)	CMI (*n* = 31)
Physical Functioning	91.67 [83.82 to 99.52]	43.50 [34.63 to 52.37]*
Role Physical	81.25 [63.03 to 99.47]	10.00 [1.02 to 18.98]*
Bodily Pain	75.75 [62.52 to 88.98	26.20 [19.82 to 32.58]*
General Health	75.33 [64.85 to 85.82]	24.83 [18.22 to 31.45]*
Vitality	62.08 [49.35 to 74.82]	14.83 [10.19 to 19.47]*
Social Functioning	82.29 [67.38 to 97.21	25.83 [18.99 to 32.68]*
Role Emotional	88.89 [74.21 to 103.57]	30.00 [15.53 to 44.47]*
Mental Health	76.67 [68.54 to 84.79]	52.40 [44.86 to 59.94]*

CMI subjects have significantly impaired quality of life. (2-tailed unpaired student’s t-test corrected using FDR (*P*<0.05): *P<0.000001 vs. Controls (Mean [95% Confidence Intervals]).

The mean dolorimetry pressure causing pain was significantly lower in CMI (3.55 kg [2.79 to 4.31]) than controls (6.73 kg [5.39 to 8.07]; *P*<0.003; Figure 1A). A multiple regression model with dolorimetry values as the dependent variable and independent covariates of Chalder’s fatigue scale, MFI general fatigue and ordinal fatigue ratings identified ordinal fatigue as the dominant predictor (*R^2^* = 0.394, β = −0.628, *F*_1,40_ = 25.4, *P* = 0.00001). Therefore, the Chalder’s fatigue scale and MFI scores were excluded from the rest of the analysis as a data reduction step.

Systemic hyperalgesia, measured by dolorimetry, was negatively correlated with McGill pain score (*R^2^* = 0.46, *P*<0.001; Figure 1B) and ordinal fatigue (*R^2^* = 0.40, *P*<0.001; Figure 1B). McGill and ordinal fatigue scores were positively correlated (*R^2^* = 0.46, *P*<0.001; Figure 1B). ROC analysis confirmed that dolorimetry, McGill total score and ordinal fatigue significantly discriminated CMI from control subjects (AUC >0.85, *P*<0.001, asymptotic significance; Figure 1C). Fatigue, pain and dolorimetry thresholds were significantly correlated with each other, and discriminated between CMI and control groups.

### DTI Parameters

**Figure 2:**
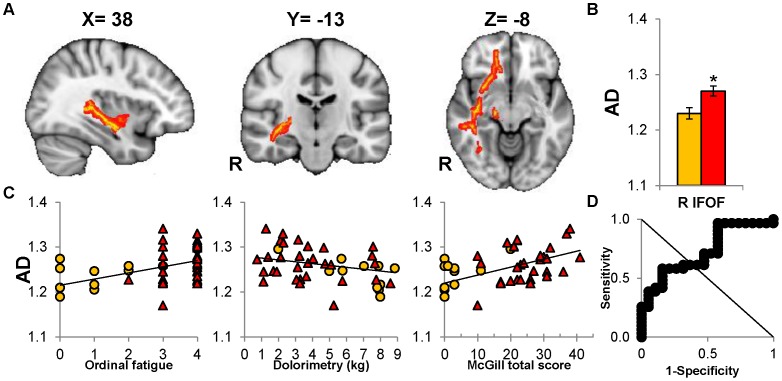
Increased axial diffusivity (AD) of right IFOF predicts CMI status. (A) Representative transverse, sagittal, and coronal views of the right IFOF (red) demonstrate projections from the prefrontal to temporo-occipital lobe. (B) CMI subjects (red) have increased AD compared to controls (orange) (*P = 0.012) (C) AD values from the combined control (orange) and CMI (red) groups significantly correlated with fatigue (*R = *0.398, *P = 0.012), dolorimetry (*R = −*0.407, *P = 0.012) and McGill total score (*R = *0.448, *P = 0.008). (D) ROC analysis for right IFOF AD confirmed the potential to discriminate between CMI and control groups (threshold = 1.24, AUC = 0.760; *P = *0.002, asymptotic significance). (*P<0.05, FDR corrected; error bar depicts ±95% confidence interval).

**Figure 3:**
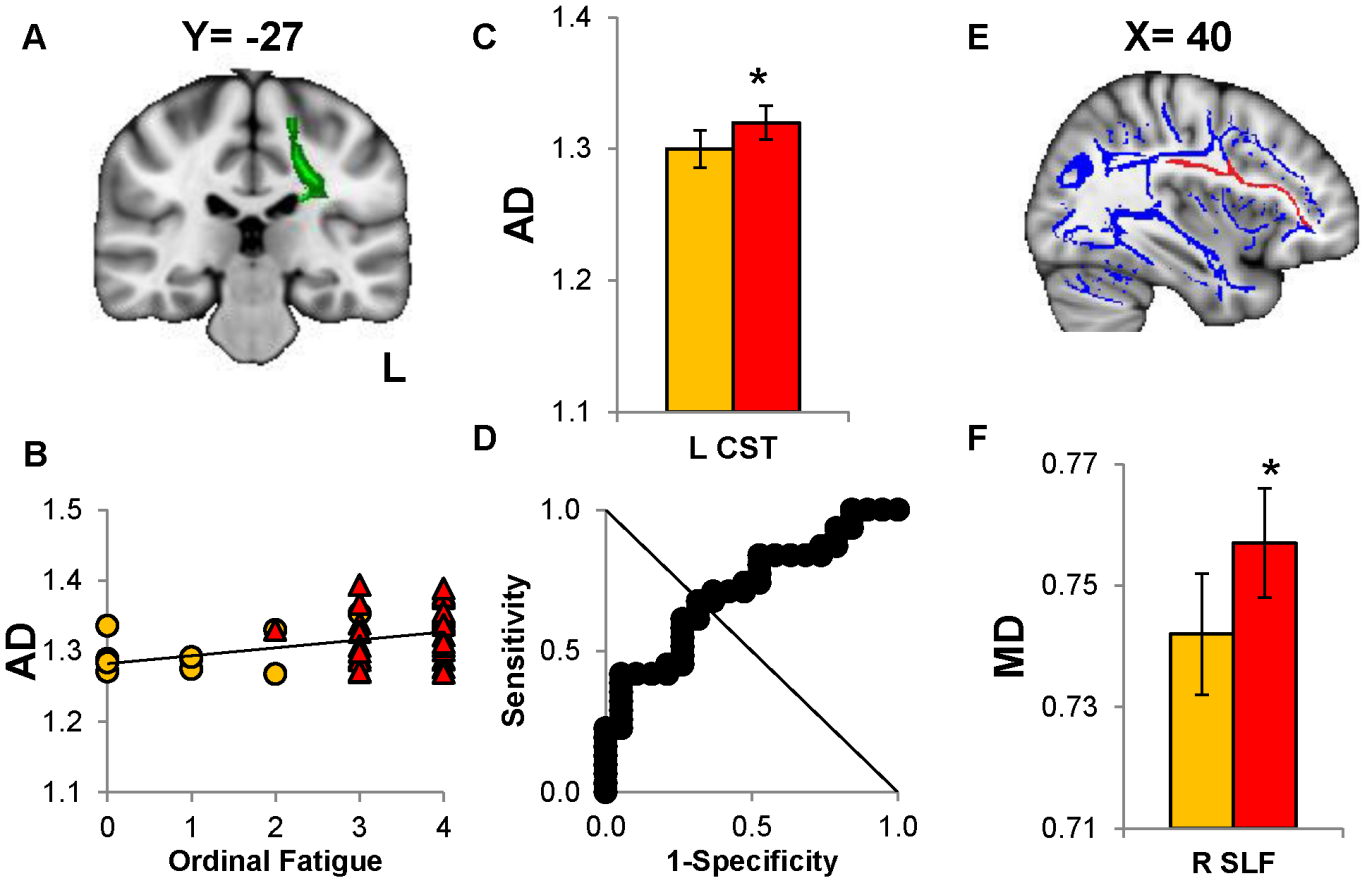
Increased mean (MD) and axial (AD) diffusivity in CMI compared to controls. (A) Coronal view of the left corticospinal tract (CST) overlaid (green) onto the MNI template. (B) AD of the left CST correlated with ordinal fatigue across all subjects (*R = *0.366, *P = 0.02). (C) The histogram depicts CMI subjects have significantly higher AD (*P = 0.047) than controls. (D) The ROC analysis confirmed the potential for AD of the left CST to distinguish CMI from controls (threshold = 1.29, AUC = 0.736; *P = *0.006, asymptotic significance). (E) Sagittal view of the right superior longitudinal fasciculus (SLF) overlaid (red) for display purposes onto the mean tract skeleton (blue). (F) The histogram indicates CMI have increased right SLF MD compared to controls (*P = 0.048). (*P<0.05, FDR corrected; error bars depict ±95% confidence interval).

A seminal finding was the significantly higher AD in CMI for the right IFOF (Figure 2A and Figure 2B) and left corticospinal tract (CST; Figure 3A and Figure 3C) for CMI compared to control subjects (Table F in File S1). MD was significantly higher in CMI subjects than controls for the right superior longitudinal fasciculus (SLF; Figure 3E, Figure 3F and Table D in File S1). FA (Table C in File S1) and RD (Table F in File S1) values were equivalent for the 2 groups.

### Clinical and DTI Correlations Across All Subjects

Fatigue and AD were positively correlated for the right IFOF (Figure 2C), right SLF, right inferior longitudinal fasciculus (ILF) and left CST (Figure 3B) across all CMI and control subjects (*P≤*0.02; Table 4).

**Table 4:** Significant correlations between clinical and diffusivity parameters for controls and CMI.

H	Tract	*r*	*R^2^*	*P* value*
**AD and dolorimetry**				
R	Cingulum	−0.437	0.191	0.008
R	IFOF	−0.407	0.165	0.012
L	IFOF	−0.381	0.145	0.015
	Forceps minor	−0.403	0.163	0.012
L	UF	−0.382	0.147	0.015
**AD and ordinal fatigue**				
R	IFOF	0.398	0.166	0.012
R	ILF	0.371	0.138	0.012
R	SLF	0.389	0.131	0.012
L	CST	0.366	0.125	0.02
**AD and McGill total score**				
R	Cingulum	0.365	0.133	0.02
R	IFOF	0.448	0.20	0.008
R	ILF	0.417	0.17	0.009
R	UF	0.375	0.14	0.018
L	UF	0.440	0.19	0.008
**MD and dolorimetry**				
L	SLF	−0.437	0.191	0.008
R	SLF	−0.456	0.208	0.004
**MD and ordinal fatigue**				
L	SLF	0.408	0.12	0.012
R	SLF	0.429	0.145	0.008
L	CST	0.364	0.067	0.02
**RD and dolorimetry**				
L	SLF	−0.402	0.10	0.012

Significant correlations between dolorimetry pain threshold, ordinal fatigue and McGill pain score with white matter properties in axial diffusivity (AD), mean diffusivity (MD), and radial diffusivity (RD). (Pearson’s and Spearman’s one-tailed correlations corrected for multiple comparisons using FDR; *P≤0.02). IFOF = inferior fronto occipital fasciculus; UF = uncinate fasciculus; ILF = inferior longitudinal fasciculus; SLF = superior longitudinal fasciculus; CST = corticospinal tract.

**Figure 4:**
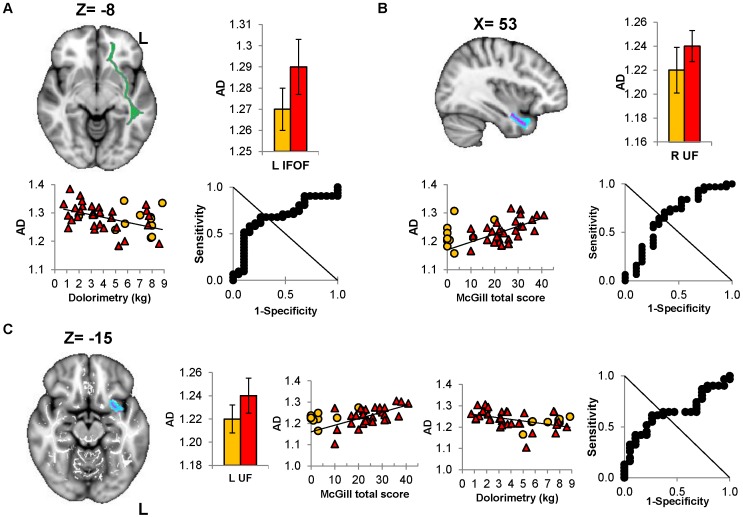
Increased axial diffusivity (AD) in left IFOF and bilateral UF predicts CMI subgroups. (A) Sagittal projection of the left IFOF (green) onto the MNI template. Individual left IFOF AD values correlated negatively with dolorimetry (*R = −*0.381, *P = 0.015). Histogram show no significant difference but ROC analysis suggest discriminatory potential for AD of the left IFOF between CMI and control groups (threshold = 1.73;AUC = 0.690, **P = 0.025). (B) The right UF (blue, transverse section) was correlated significantly with McGill total score (*R = *0.375, *P = 0.018). Histogram shows no significant difference but ROC analysis suggested potential to distinguish CMI from controls groups (threshold = 1.22; AUC = 0.707, **P = 0.016). (C) The left UF (blue, sagittal view) had AD values that significantly correlated with dolorimetry (*R = −*0.382, *P = 0.015) and McGill total score (*R = −*0.440, *P = 0.008). No significant difference in AD for the left UF but ROC analysis of the AD values confirmed its discriminatory potential (threshold = 1.23; AUC = 0.682, **P = 0.034). (*P<0.05, FDR corrected;**P<0.05, asymptotic significance; error bars depict ±95% confidence interval) IFOF = inferior fronto occipital fasciculus UF = uncinate fasciculus.

Dolorimetry pressures and AD were inversely correlated for the right IFOF (Figure 2C), left IFOF (Figure 4A), left uncinate fasciculus (UF), right cingulum, and forceps minor (Table 4). The McGill pain score correlated with AD in the right IFOF (Figure 2C), left and right UF (Figure 4B and 4C), right cingulum and right ILF (Table 4).

Dolorimetry was inversely correlated with MD in the left and right SLF, and RD in the left SLF (Table 4; *P≤*0.02). Ordinal fatigue and MD were positively correlated for the left CST and bilateral SLF (Table 4; *P≤*0.02). FA had no significant correlations with clinical scores.

Multiple regression models using the clinical variables as dependent values incorporated covariates of age, gender, and the AD, MD and RD values from those tracts with significant correlates (Table 4). AD in the right IFOF was the dominant predictor of both ordinal fatigue (*R^2^* = 0.159, β = 0.399, *F*_1,40_ = 7.58, *P = *0.009) and McGill pain score (*R^2^* = 0.200, β = 0.448, *F*_1,40_ = 9.78, *P = *0.003). The regression model for dolorimetry identified AD of the right cingulum (β = −0.394, *P = *0.006) and right IFOF (β = −0.329, *P = *0.02) as significant predictors of tenderness (*R^2^* = 0.297, *F*_2,39_ = 8.25, *P = *0.001). AD of the right IFOF was consistently associated with fatigue, pain, and hyperalgesia.

### CMI versus Control Status

ROC analysis of AD values identified 5 tracts that distinguished CMI from control subjects: right IFOF (specificity 52.6%, sensitivity 71.7%, AUC = 0.760; Figure 2D), left CST (specificity 68.4%, sensitivity 67.7%, AUC = 0.736; Figure 3D), left IFOF (specificity 73.7%, sensitivity 67.7%, AUC = 0.693; Figure 4A), right UF (specificity 63.2%, sensitivity 71.1%, AUC* = *0.707; Figure 4B) and left UF (specificity 73.7%, sensitivity 61.3%, AUC = 0.682; Figure 4C). FA, RD and MD did not discriminate between groups.

Of the 5 tracts, CMI subjects’ dolorimetry pressures were negatively correlated with the right IFOF(*r = *−0.453, *P = *0.015) and left UF (*r = *−0.423, *P = *0.018). McGill total scores correlated with the left (*r = *0.565, *P = *0.004) and right UF (*r = *0.572, *P = *0.002). In control subjects, dolorimetry pressures were negatively correlated with the right IFOF (*r = *−0.679, *P = *0.02); significance was lost when a single outlier was removed (*r = *−0.467, *P = *0.174). McGill scores and ordinal fatigue did not correlate with DTI parameters in controls.

Separate multivariate regression models in the CMI and control groups used dolorimetry pressures as the dependent variable incorporated covariates of age, gender, McGill total score, fatigue rating, and the AD values from the 5 tracts with significant ROC analysis. For CMI subjects, AD of the right IFOF was the dominant predictor of dolorimetry pressure (*R^2^* = 0.186, β = − 0.432, *F*_1,29_ = 6.42, *P = *0.017). For control subjects, McGill total scores were predominant (*R^2^* = 0.632, β = 0.795, *F*_1,10_ = 15.43, *P = *0.003). This indicated that AD in the right IFOF was the primary contributor to tenderness measured by dolorimetry in CMI patients, all with clinically significant pain. However, the right IFOF played no role in predicting tenderness in patients without clinically significant pain.

### Binary Analysis

Binary logistic regression used CMI and control status to identify the most significant tracts that distinguished between the 2 groups. The independent variables were the diffusivity measures of the 7 tracts identified by significant ROC and multiple regression analyses: AD for the left and right IFOF, left and right UF, right cingulum, and left CST, and MD for right SLF. CMI status was significantly predicted by AD of the right IFOF (χ^2^ = 9.559, *P* = 0.002). Accuracy of the model was 0.66 (odds ratio of 1.028, 95% CI [1.008 to 1.049]).

## Discussion

All of the veterans recruited for this protocol met CMI and CFS criteria [2], [5]. To understand the pathophysiological principles behind these case designation criteria, we examined the underlying complaints that were most strongly reported by our subjects. Dolorimetry, fatigue, and pain ratings were highly correlated with each other and with elevated AD in cortico-cortical association and corticospinal tracts.

These analyses identified four significant correlates of CMI status that were significantly different from controls: ordinal fatigue, McGill total pain scores, dolorimetry (kg) and AD of the right IFOF. The salutary observation was that CMI status was associated with increased axial diffusivity in the right IFOF with non-significant trends for increased FA and MD in the same tract (Table C in File S1) (Table D in File S1). Multi-variate and binary logistic regression analysis identified the right IFOF as the only tract to correlate with all three clinical parameters and may provide diagnostic utility in predicting CMI versus control status.

The right IFOF connects multiple frontal, sublobar, temporal and occipital cortical regions that are involved in the perception of pain, fatigue and cognitive dysfunction that are symptom constructs in the case designation criteria for CMI [2] and CFS [5]. Anatomically, the tract originates from the vmPFC, inferior frontal gyrus, frontal pole and OFC [59]. As it leaves the prefrontal area it courses adjacent to the insula [60] and through the temporal lobe to terminate in the posterior fusiform, cuneus, and lateral cortices of the occipital lobe [59].

The OFC and vmPFC are intimately associated with the severity of fatigue [10], [16] and communicate with the nucleus accumbens via corticostriatal IFOF fibers [18]. During the onset of noxious stimuli these regions coordinate responses that provide a punishing teaching signal that leads to altered decision making based upon these painful cues [61]. Increased structural connectivity between the regions linked by the right IFOF is predictive of increased blood flow to the nucleus accumbens which represents amplified sensitivity to punishment during reward-related behavior [18]. Sensory and other processing through the anterior insula directly contributes to the vmPFC - nucleus accumbens interactions which are causally associated with the transition from subacute to chronic low back pain [19]. Because the right IFOF is a critical component of the structural circuitry that facilitates communication between these regions, increased AD in the right IFOF may play a central role in the adaptation and maintenance of chronic pain and fatigue in CMI.

Attention and focus are other functions mediated via the right IFOF. This tract connects the right inferior frontal gyrus and right temporal parietal junction that form the ventral attention network (VAN) [62]. VAN maintains surveillance for unexpected environmental cues that may be salient. Maintaining focus on goal – directed behavior is the function of the dorsal attention network (DAN) [63]. Anatomically, DAN includes the frontal eye fields, supplementary motor cortex and dorsolateral prefrontal cortex. DAN generates top – down managerial control to complete specific tasks [63]. Activation of VAN creates an interruptive signal that decouples DAN activity so that attention must be reoriented towards the newly identified, task – relevant stimulus. Increased VAN activity has been linked to decreased activity in the DAN [64].

Nociceptive stimuli can involuntarily capture attention and interfere with on – going behavior [65], [66]. Building on this notion, elevated AD in the right IFOF in CMI may signify increased structural connectivity with a heightened propensity to activate VAN, interrupt DAN activity, and cause reorientation of attention to pain signals. Increased connectivity may explain the reported surveillance and hyperarousal behaviors of CMI subjects [1], [67].

The left CST and right SLF had higher diffusivity measures between CMI and control groups. Increased AD in the left CST, which sends collaterals into the spinal cord dorsal horn, may suggest potential dysfunction of descending anti-nociceptive pathways that may contribute to hyperalgesia [68]. MD was increased in the right SLF and correlated with greater fatigue and lower dolorimetry pain pressures. This tract connects the dorsolateral prefrontal cortex and DAN to inferior parietal working memory regions [69]. This is of relevance to CMI since increased MD in the SLF has been correlated with language impairment and cognitive deficits [70], [71].

Increased AD was correlated with fatigue and pain measures in several other tracts when all subjects were assessed. The right cingulum links the anterior and posterior cingulate gyrus and was associated with nociceptive processing in several chronic pain states [9], [12], [72]. Elevated AD was correlated with the McGill total score and lower dolorimetry pressures. The forceps minor connects regions in the left and right anterior prefrontal lobes that are implicated in pain and fatigue processing [10], [13], [16], [17], [19].The uncinate fasciculus links prefrontal and limbic regions involved in pain, emotion and affect [73]. The increase and discriminatory potential in AD suggests DTI analysis may have value as a research tool to identify longitudinal changes and to test the efficacy of novel therapies in clinical trials in CMI.

This pilot study has several limitations. The magnitudes of the differences in AD between groups were not large, but were statistical significant after correction for multiple comparisons. Correlations of AD for various tracts with fatigue, pain and dolorimetry measures identified significant relationships within the entire study population.

The control group was not entirely Gulf War veterans who shared the same exposures and experiences as the CMI subjects. However, our subjects (Table A in File S1) were representative of the 1995 Gulf War National Health Survey and other population based studies [1], [6], [74]. Prospective epidemiological studies using the variables identified here will be required to firmly resolve this issue.

Correlations between the increased AD in right IFOF, fatigue, pain, and hyperalgesia may have been an artifact of selecting subjects with similar complaints who represented a distal end of a spectrum found in the general population. Our sample size was not large enough to adequately assess this possibility. However, the binary logistic regression analysis significantly distinguished CMI from control subjects. This suggests a bimodal rather than unimodal distribution.

Tractography and analysis of gray matter volume loss in regions linked by the dysfunctional white matter tracts were not investigated, but may provide important information about the heterogeneity and extent of CMI dysfunction. Reduced midbrain white matter volume was correlated with duration of fatigue in CFS [75], but DTI was not performed. Tractography offers a complementary approach to TBSS. TBSS may underestimate DTI indices since it relies on the white matter skeleton with highest FA values [76].

Axonal atrophy may lead to an artifactual increase in AD since smaller axonal caliber may increase absolute neuron densities within pixels that are mathematically transformed into higher diffusivity measurements when mapped onto a standard white matter skeleton [77]. TBSS processing as used here may reduce this potential bias [76]. This and other explanations for elevated AD await histological verification from surgical or autopsy studies of GWI veterans.

The cross - sectional design cannot address longitudinal changes or temporal reproducibility. Future longitudinal DTI studies will be needed to confirm if the defect of elevated AD correlates with fatigue, pain and hyperalgesia as it changes over time.

### Conclusion

The right inferior fronto-occipital fasciculus links cortical regions involved in fatigue, pain, emotional and reward processing, and the right ventral attention network in cognition. Axial diffusivity of this region was significantly different between CMI and controls and the degree of difference was found to correlate to fatigue and pain symptoms. The axonal neuropathological mechanism(s) explaining the objectively measured increase in axial diffusivity may contribute to Gulf War Illness.

## Supporting Information

File S1 **Supporting information tables.** Table A: Extended and detailed demographics information. Table B: Detailed recruitment strategies and retention during protocol. Table C: Average fractional anisotropy (FA) for 20 white matter tracts between groups. Table D: Average mean diffusivity (MD) for 20 white matter tracts between groups. Table E: Average axial diffusivity (AD) for 20 white matter tracts between groups. Table F: Average radial diffusivity (RD) for 20 white matter tracts between groups.
(DOCX)
