# Supplementary material for: Increased Brain White Matter Axial Diffusivity Associated with Fatigue, Pain and Hyperalgesia in Gulf War Illness
Source: PLoS One. 2013 Mar 20;8(3):e58493. doi: 10.1371/journal.pone.0058493 (PMC3603990; doi:10.1371/journal.pone.0058493)
Supplement: File S1 — Supporting information tables. Table A: Extended and detailed demographics information. Table B: Detailed recruitment strategies and retention during protocol. Table C: Average fractional anisotropy (FA) for 20 white matter tracts between groups. Table D: Average mean diffusivity (MD) for 20 white matter tracts between groups. Table E: Average axial diffusivity (AD) for 20 white matter tracts between groups. Table F: Average radial diffusivity (RD) for 20 white matter tracts between groups. (DOCX) [file pone.0058493.s001.docx]

Supporting Online Material for

Increased brain white matter axial diffusivity is associated with pain, fatigue and hyperalgesia in Gulf War Illness

Rakib U. Rayhan, M.S. ^1*^ Benson W. Stevens, B.S. ^2^ Christian R. Timbol, M.S. ^1^Toyin Adewuyi, M.S.^1^ Brian T. Walitt M.D.^3^ John VanMeter, Ph.D. ^2^ James N. Baraniuk, M.D.^1^

^1^ Division of Rheumatology, Immunology and Allergy; Department of Medicine, Georgetown University Medical Center Room 3004F, 3rd Floor PHC Building, 3800 Reservoir Road, NW

Washington, DC 20007-2197 ^2^ Center for Functional and Molecular Imaging, Georgetown University Medical Center, Suite LM14, Preclinical Sciences Building, 3900 Reservoir Road,NW, Washington, DC 20007-2197. ^3^ Division of Rheumatology, Washington Hospital Center, 110 Irving Street, NW Washington, DC 20010 TEL: 202-877-7000, E-mail:brian.t.walitt@medstar.net

*To whom correspondence should be addressed: Electronic mail: rur@georgetown.edu

Supporting Online Material Contains:

2. Tables A-F

|  | **Controls**  **(Initial DTI)*** | **Controls**  **(Correlation analysis)** | **CMI** |
| --- | --- | --- | --- |
| *N* | 20 | 12 | 31 |
| **Age**  mean [95% C.I.] | 45.6  [41.2 to 50.5] | 47.5  [43.2 to 53.9] | 45.9  [43.2 to 48.4] |
| **Gender** |  |  |  |
| Male | 11 | 11 | 25 |
| Female | 9 | 1 | 6 |
| **Race** |  |  |  |
| White | 16 | 11 | 21 |
| Black | 3 | 0 | 9 |
| Other | 1 | 1 | 1 |
| **Branch** |  |  |  |
| Air Force | 4 | 4 | 7 |
| Army | 3 | 3 | 18 |
| Marines | 2 | 2 | 3 |
| Navy | 0 | 0 | 3 |
| Civilian | 11 | 3 | 0 |
| **Rank at discharge** |  |  |  |
| Enlisted | 7 | 7 | 25 |
| Officers | 2 | 2 | 6 |
| **Exposures** |  |  |  |
| Theater |  | 7 / 12 (58%) | 25 / 31 (81%) |
| SCUD missile attack |  | 5 / 12 (42%) | 16 / 31 (52%) |
| Combat |  | 4 / 12 (33%) | 17 / 31 (55%) |
| **Khamisiyah** |  | 2 / 12 (17%) | 7 / 28 (25%) |
| Chemical alarms |  | 5 / 12 (42%) | 20 / 31 (65%) |
| Oil fires and smoke |  | 3 / 12 (25%) | 20 / 31 (65%) |
| Volatile organic compounds** |  | 2 / 12 (17%) | 23 / 31 (74%) |
| Vaccinations |  | 7 / 12 (58%) | 31 / 31 (100%) |
| Tinnitus |  | 5 / 12 (42%) | 17 / 31 (55%) |
| Sleep apnea |  | 1 / 12 (8%) | 9 / 22 (41%) |
| Cognitive behavioral therapy |  | 4 / 12 (33%) | 25 / 31 (81%) |
| Chronic Fatigue Syndrome | 0% | 0 / 12 (0%) | 31 / 31 (100%) |
| Fibromyalgia | 10% | 2 / 11 (17%) | 15 / 29 (52%) |

**Table A. Extended demographics**

* Eight control civilians were included in the initial exploratory DTI analysis. They did not complete the entire questionnaire or dolorimetry analysis so their data were not used for the correlation analysis.

** Direct contact with >10% of skin surface and inhalation of liquid diesel, jet fuels, hydraulic fluids, paints or other volatile organic compounds for more than 30 days.

|  | Recruitment of Gulf War Veterans and sedentary civilians |
| --- | --- |
| Study Approvals | This protocol was approved by the Georgetown University Institutional Review Board (IRB #2009-229) and USAMRMC Human Research Protection Office (HRPO #A-15547.0) (clinicaltrials.gov identification number NCT01291758). Additional civilians were recruited for DTI only from IRB #2010-050 and #2010-356. |
| Recruitment | 2009 to 2011: Print and on-line advertising, word-of-mouth and internet contacts through support groups and personal contacts |
| Potential candidates | N = 250 healthy, sedentary and GWI veterans contacted the laboratory  N = 23 sedentary civilians to augment control group sample size |
| Action | Scripted, confidential telephone screening with description of the study. Provisional informed consent. Assessment of inclusion and exclusion criteria. |
| Provisional subject selection | Inclusion for Veterans: Active military duty between August 1, 1990 and July 31, 1991, and deployment for ≥ 30 consecutive days to the Persian Gulf War region.  CMI criteria required ≥ 6 months of complaints from at least two of the following categories: (i) fatigue; (ii) musculoskeletal (joint pain, joint stiffness or muscle pain); and (iii) mood and cognition (feeling depressed, trouble remembering or focusing, mood swings, feeling anxious, difficulty finding words, and difficulty sleeping) [2].  CFS criteria required significant disability due to fatigue that was not improved by refreshing sleep; plus significant complaints in 4 of the following 8 categories: memory and concentration, sore throat, sore lymph node regions, myalgia, arthralgia, headaches, sleep disturbances, and exertional exhaustion [5]. Inclusion for Civilians: Currently sedentary lifestyle. Several had extensive immunizations and work experience in the Persian Gulf region or other high risk environments that were comparable to Veterans. Exclusion: Currently active duty military personnel; claustrophobia; intolerance of needles; major psychiatric illness; cardiovascular limitations; infectious (e.g. HIV), neoplastic, untreated endocrine or other chronic diseases that may have accounted for CMI and CFS associated symptoms. Exclusion from fMRI because of ferrous based implants. Highly active lifestyle. |
| Loss of candidates | N = 188 (N = 149 declined to participate; N = 39 failed to respond to further inquiries) |
| Eligible candidates | N = 66 healthy and GWI veterans. N = 19 civilians. |
| On-line questionnaires | Subjects received a confidential website log-in identifier, password and identification code. They completed the pre-study symptom and psychometric questionnaires using our eZhengtricity data collection process. No personal identifying information was collected using this system. (http://www9.georgetown.edu/faculty/baraniuj/Site/Ezhengtricity.html) |
| Entered Clinical Research Unit protocol | Written informed consent.History and physical examination to confirm criteria for GWI, CFS and other diagnoses.Blood work for HIV, pregnancy, complete blood count, electrolytes, transaminases, thyroid, renal function, sedimentation rate, antinuclear antibody, rheumatoid factor and other tests to identify additional exclusionary conditions. |
| Loss of subjects | N = 34 (N = 15 medical exclusion; N = 12 equipment or other technical failures; N = 7 no show, failed to respond to further inquiries) |
| DTI and clinical correlation analysis | CMI + CFS group: N = 31 sedentary veterans meeting both CMI + CFS criteria  Control group: N =1 sedentary healthy veteran; N = 2 veterans with fibromyalgia but not meeting CMI or CFS criteria; N = 9 age and gender matched sedentary civilian controls with no exclusion criteria and not meeting CFS criteria.Sum = 12 controls with history, physical, questionnaires and fMRI but not meeting CMI + CFS criteria  Additional Controls for initial DTI analysis only:  N = 8 additional age matched sedentary control females added solely for initial fMRI analysis (IRB #2010-050 and #2010-356).  Sum = 20 control subjects not meeting CMI + CFS criteria. |

**Table B. Recruitment and retention during protocol.**

| **H** | **Tract** | **Controls (n=20)** | **CMI (n=31)** |
| --- | --- | --- | --- |
| L | Superior cerebellar peduncle | 0.538 [0.524 to 0.552] | 0.564 [0.548 to 0.581] |
| R | Superior cerebellar peduncle | 0.564 [0.548 to 0.581] | 0.573 [0.534 to 0.613] |
| L | Anterior thalamic radiation | 0.435 [0.423 to 0.446] | 0.436 [0.427 to 0.444] |
| R | Anterior thalamic radiation | 0.389 [0.379 to 0.398] | 0.389 [0.380 to 0.397] |
| L | Cingulum cingulate | 0.619 [0.596 to 0.642] | 0.621 [0.597 to 0.644] |
| R | Cingulum cingulate | 0.486 [0.464 to 0.507] | 0.487 [0.472 to 0.502] |
| L | Cingulum hippocampus | 0.415 [0.401 to 0.430] | 0.423 [0.410 to 0.435] |
| R | Cingulum hippocampus | 0.413 [0.396 to 0.430] | 0.408 [0.396 to 0.420] |
| L | Corticospinal tract | 0.585 [0.575 to 0.594] | 0.590 [0.580 to 0.600] |
| R | Corticospinal tract | 0.600 [0.588 to 0.611] | 0.604 [0.594 to 0.614] |
|  | Forceps major | 0.662 [0.646 to 0.677] | 0.664 [0.652 to 0.675] |
|  | Forceps minor | 0.535 [0.524 to 0.547] | 0.541 [0.531 to 0.551] |
| L | Inferior fronto-occipital fasciculus | 0.452 [0.439 to 0.465] | 0.456 [0.448 to 0.463] |
| R | Inferior fronto-occipital fasciculus | 0.456 [0.442 to 0.470] | 0.467 [0.457 to 0.476] |
| L | Inferior longitudinal fasciculus | 0.490 [0.477 to 0.502] | 0.496 [0.485 to 0.507] |
| R | Inferior longitudinal fasciculus | 0.541 [0.526 to 0.557] | 0.554 [0.540 to 0.569] |
| L | Superior longitudinal fasciculus | 0.479 [0.468 to 0.491] | 0.471 [0.459 to 0.482] |
| R | Superior longitudinal fasciculus | 0.507 [0.499 to 0.516] | 0.496 [0.484 to 0.508] |
| L | Uncinate fasciculus | 0.415 [0.405 to 0.426] | 0.415 [0.405 to 0.425] |
| R | Uncinate fasciculus | 0.408 [0.398 to 0.418] | 0.405 [0.396 to 0.415] |

**Table C. Average fractional anisotropy (FA) values.** CMI subjects and controls have similar fractional anisotropy values. H*=* Hemisphere (Mean [95% Confidence intervals])

| **H** | **Tract** | **Controls (n=20)** | **CMI (n=31)** |
| --- | --- | --- | --- |
| L | Superior Cerebellar Peduncle | 1.15 [1.11 to 1.19] | 1.14 [1.06 to 1.21] |
| R | Superior Cerebellar Peduncle | 1.04 [1.01 to 1.08] | 1.04 [0.97 to 1.11] |
| L | Anterior thalamic radiation | 0.763 [0.748 to 0.777] | 0.773 [0.758 to 0.786] |
| R | Anterior thalamic radiation | 0.802 [0.785 to 0.820] | 0.814 [0.798 to 0.831] |
| L | Cingulum cingulate | 0.774 [0.762 to 0.786] | 0.788 [0.775 to 0.800] |
| R | Cingulum cingulate | 0.779 [0.764 to 0.794] | 0.785 [0.771 to 0.798] |
| L | Cingulum hippocampus | 0.758 [0.741 to 0.774] | 0.761 [0.749 to 0.774] |
| R | Cingulum hippocampus | 0.773 [0.752 to 0.795] | 0.775 [0.764 to 0.786] |
| L | Corticospinal tract | 0.740 [0.730 to 0.749] | 0.749 [0.741 to 0.757] |
| R | Corticospinal tract | 0.744 [0.733 to 0.755] | 0.747 [0.740 to 0.754] |
|  | Forceps major | 0.837 [0.798 to 0.876] | 0.847 [0.813 to 0.882] |
|  | Forceps minor | 0.798 [0.783 to 0.813] | 0.797 [0.786 to 0.808] |
| L | Inferior fronto-occipital fasciculus | 0.824 [0.807 to 0.841] | 0.834 [0.822 to 0.847] |
| R | Inferior fronto-occipital fasciculus | 0.795 [0.784 to 0.806] | 0.805 [0.793 to 0.816] |
| L | Inferior longitudinal fasciculus | 0.802 [0.792 to 0.812] | 0.793 [0.764 to 0.822] |
| R | Inferior longitudinal fasciculus | 0.781 [0.771 to 0.791] | 0.785 [0.774 to 0.796] |
| L | Superior longitudinal fasciculus | 0.748 [0.736 to 0.760] | 0.762 [0.751 to 0.773] |
| R | Superior longitudinal fasciculus | 0.742 [0.731 to 0.752] | 0.757 [0.747 to 0.767] * |
| L | Uncinate fasciculus | 0.821 [0.807 to 0.835] | 0.830 [0.818 to 0.842] |
| R | Uncinate fasciculus | 0.822 [0.807 to 0.838] | 0.839 [0.825 to 0.853] |

**Table D. Average mean diffusivity (MD) values.** CMI subjects had a significantly higher MD value in the right superior longitudinal fasciculus (**P* =0.048) compared to controls. Significant *P* values identified by 2-tailed unpaired student’s t-test corrected using FDR (*P<*0.05) [95% Confidence intervals]; H*=* Hemisphere

| **H** | **Tract** | **Controls (n=20)** | **CMI (n=31)** |
| --- | --- | --- | --- |
| L | Superior Cerebellar Peduncle | 1.83 [1.78 to 1.88] | 1.83 [1.74 to 1.92] |
| R | Superior Cerebellar Peduncle | 1.74 [1.68 to 1.79] | 1.74 [1.65 to 1.82] |
| L | Anterior thalamic radiation | 1.14 [1.12 to 1.16] | 1.16 [1.14 to 1.17] |
| R | Anterior thalamic radiation | 1.14 [1.12 to 1.16] | 1.16 [1.14 to 1.18] |
| L | Cingulum cingulate | 1.41 [1.37 to 1.44] | 1.44 [1.40 to 1.47] |
| R | Cingulum cingulate | 1.24 [1.22 to 1.26] | 1.25 [1.23 to 1.26] |
| L | Cingulum hippocampus | 1.11 [1.09 to 1.14] | 1.13 [1.11 to 1.15] |
| R | Cingulum hippocampus | 1.13 [1.11 to 1.15] | 1.13 [1.11 to 1.15] |
| L | Corticospinal tract | 1.30 [1.28 to 1.31] | 1.32 [1.31 to 1.34] * |
| R | Corticospinal tract | 1.33 [1.31 to 1.35] | 1.34 [1.32 to 1.35] |
|  | Forceps major | 1.58 [1.51 to 1.65] | 1.60 [1.56 to 1.64] |
|  | Forceps minor | 1.33 [1.31 to 1.35] | 1.34 [1.32 to 1.35] |
| L | Inferior fronto-occipital fasciculus | 1.26 [1.24 to 1.28] | 1.28 [1.27 to 1.30] |
| R | Inferior fronto-occipital fasciculus | 1.23 [1.22 to 1.24] | 1.27 [1.25 to 1.28] ** |
| L | Inferior longitudinal fasciculus | 1.26 [1.25 to 1.28] | 1.28 [1.26 to 1.31] |
| R | Inferior longitudinal fasciculus | 1.30 [1.28 to 1.33] | 1.33 [1.31 to 1.35] |
| L | Superior longitudinal fasciculus | 1.15 [1.13 to 1.16] | 1.16 [1.14 to 1.17] |
| R | Superior longitudinal fasciculus | 1.17 [1.16 to 1.19] | 1.18 [1.17 to 1.20] |
| L | Uncinate fasciculus | 1.22 [1.21 to 1.23] | 1.23 [1.22 to 1.25] |
| R | Uncinate fasciculus | 1.22 [1.20 to 1.24] | 1.23 [1.22 to 1.25] |

**Table E. Average axial diffusivity (AD) values.** AD was significantly higher for CMI than controls for the left corticospinal tract (* *P* = 0.047) and right inferior fronto-occipital fasciculus (** *P* =0.015) Significant *P* values identified by 2-tailed unpaired student’s t-test corrected using FDR (*P<*0.05). (Mean [95% Confidence intervals]; H*=* Hemisphere)

| **H** | **Tract** | **Controls (n=20)** | **CMI (n=31)** |
| --- | --- | --- | --- |
| L | Superior Cerebellar Peduncle | 0.806 [0.771 to 0.840] | 0.791 [0.708 to 0.873] |
| R | Superior Cerebellar Peduncle | 0.696 [0.661 to 0.730] | 0.683 [0.606 to 0.761] |
| L | Anterior thalamic radiation | 0.571 [0.557 to 0.586] | 0.577 [0.562 to 0.592] |
| R | Anterior thalamic radiation | 0.630 [0.612 to 0.649] | 0.639 [0.622 to 0.656] |
| L | Cingulum cingulate | 0.455 [0.431 to 0.479] | 0.461 [0.437 to 0.485] |
| R | Cingulum cingulate | 0.547 [0.523 to 0.571] | 0.552 [0.533 to 0.571] |
| L | Cingulum hippocampus | 0.578 [0.560 to 0.596] | 0.575 [0.560 to 0.590] |
| R | Cingulum hippocampus | 0.594 [0.568 to 0.620] | 0.597 [0.586 to 0.608] |
| L | Corticospinal tract | 0.459 [0.448 to 0.471] | 0.462 [0.451 to 0.473] |
| R | Corticospinal tract | 0.449 [0.436 to 0.462] | 0.449 [0.439 to 0.460] |
|  | Forceps major | 0.463 [0.431 to 0.495] | 0.468 [0.435 to 0.501] |
|  | Forceps minor | 0.528 [0.511 to 0.544] | 0.527 [0.515 to 0.539] |
| L | Inferior fronto-occipital fasciculus | 0.604 [0.583 to 0.624] | 0.609 [0.595 to 0.622] |
| R | Inferior fronto-occipital fasciculus | 0.576 [0.560 to 0.591] | 0.575 [0.562 to 0.589] |
| L | Inferior longitudinal fasciculus | 0.569 [0.555 to 0.582] | 0.564 [0.551 to 0.578] |
| R | Inferior longitudinal fasciculus | 0.518 [0.504 to 0.531] | 0.509 [0.494 to 0.525] |
| L | Superior longitudinal fasciculus | 0.545 [0.530 to 0.561] | 0.563 [0.548 to 0.578] |
| R | Superior longitudinal fasciculus | 0.524 [0.511 to 0.537] | 0.542 [0.527 to 0.556] |
| L | Uncinate fasciculus | 0.620 [0.603 to 0.637] | 0.627 [0.613 to 0.640] |
| R | Uncinate fasciculus | 0.623 [0.607 to 0.640] | 0.640 [0.623 to 0.657] |

**Table F. Average radial diffusivity (RD) values.** There were no significant differences in RD parameters between CMI and control subjects. (Mean [95% Confidence intervals]; H*=* Hemisphere)
